# The Perceived Social Stigma of People with Epilepsy with regard to the Question of Employability

**DOI:** 10.1155/2018/4140508

**Published:** 2018-05-13

**Authors:** Jéssica Lopes de Souza, Aline Scardoeli Faiola, Carmen Silvia Molleis Galego Miziara, Maria Luiza Giraldes de Manreza

**Affiliations:** ^1^Department of Legal Medicine, ABC Faculty of Medicine, Medical School Foundation, Sao Paulo, SP, Brazil; ^2^Department of Neurology, Hospital das Clínicas da Faculdade de Medicina da Universidade de São Paulo, São Paulo, SP, Brazil

## Abstract

**Objective:**

To evaluate the perceived social stigma of people with epilepsy with regard to the question of employability.

**Methods:**

A structured questionnaire was given to two groups of people with chronic diseases: those with epilepsy (study group) and those with heart disease (control group). The questions concerned employability.

**Results:**

Having epilepsy was more strongly associated with higher unemployment rates (*p* < 0.0001); job layoffs (*p* = 0.001); being unfit to work (*p* < 0.0001); feeling shame for having the disease (*p* = 0.014); absence of partners (*p* = 0.026); and depression (*p* = 0.004). The tendency to hide their disease from their employers was similar for the two groups.

**Conclusion:**

The age discrepancy between groups was an important limiting factor of this study. However, despite the limited number of participants and the age difference between the groups, there is no impediment in stating that people with epilepsy show high rates of unemployment, depression, and stigma.

## 1. Introduction

Epilepsy is a very common chronic disease; it is estimated that more than 65 million people have epilepsy worldwide [1]. One person in 26 will develop epilepsy at some point in life [2]. Three-quarters of those affected live in low- and middle-income countries [3]. Studies in Brazil showed that, in 2006, the prevalence of epilepsy in the country was 9.2/1.000 inhabitants. In 1986, an estimated prevalence of 11.9 per 1.000 inhabitants was reported for the city of São Paulo [4]. In the 21st century, people with epilepsy still suffer prejudice and stigma even though approximately 70% of people with epilepsy (PWE) control their seizures with appropriate antiepileptic medication (AEM) [5]. Because seizures are unpredictable and do not remain hidden in the social environment, epilepsy is a disruptive condition in all social situations and strongly interferes with employability. Unlike other chronic diseases, the clinical manifestations of epilepsy are highly visible, leading to greater barriers and lower social acceptability [6]. Consequently, PWE have higher rates of unemployment and underemployment [7]. A study comparing employability between PWE and people without epilepsy showed that only 39% of PWE were employed full time, compared to 55% of those without epilepsy, and that 33% were unemployed, compared to 13% in the control group [8]. The rate of unemployment and underemployment among PWE exceeds that of the general population [9]. In addition, PWE often have greater difficulty keeping their jobs [10]. Thus, epilepsy imposes a high social and personal burden on PWE, especially in those who are of working age [11].

So and Penry, 1981, confirmed in their study that the unemployment rate of PWE is twice that of the general population and that 50% of workers do not disclose their epilepsy to potential employers when applying for jobs. Many employers refuse to hire workers with epilepsy because they believe they are more susceptible to accidents and absenteeism from work [12, 13]. Job restrictions imposed on PWEs, in most cases, are unfair and based on prejudices and stereotypes [14]. The rate of accidents at work is not higher in PWEs compared to people without epilepsy [15]. Epilepsy is not the only condition that can result in sudden loss of consciousness. People with cardiogenic or metabolic syncope can also experience these symptoms. Nevertheless, the effects of superstition, stigma and prejudice make epilepsy a contributor to socioeconomic deprivation for many PWEs, due to its negative impact on employability [16]. This study aimed to estimate the perception of people with epilepsy in relation to social stigma, with a focus on the issue of employability.

## 2. Materials and Methods

50 patients with epilepsy (study group) and 50 patients with heart disease (control group) attending at the outpatient clinics of the Faculdade de Medicina do ABC were screened. Patients were interviewed using a structured protocol composed of demographic, clinical, and patient-reported information about employment. The data were obtained in 2016. This project was approved by the Ethics and Research Committee of the Faculty of Medicine at ABC Medical School.

The* inclusion criteria* were as follows: (1) a diagnosis of epilepsy based on the International League Against Epilepsy or a diagnosis of heart disease based on the Brazilian Society of Cardiology; (2) age 18–65 years; (3) both men and women were included; (4) an educational level higher than primary school, and (5) signed informed consent.

For participants with epilepsy, additional inclusion criteria included the following: clinical manifestations compatible with epileptic seizures; at least one interictal electroencephalogram examination with epileptiform discharges; more than one seizure per month (either focal with or without generalization or generalized seizure) in the last 12 months; use of one or two antiepileptic medications.

For participants with heart disease (heart failure), the additional criteria included were clinical manifestations, classes II or III according to the New York Heart Association (NYHA) functional classification (NYHA); ejection fraction higher than 50%; and no use of inotropic medication.

The* exclusion criteria* were as follows: (1) progression of central nervous system disease or acute stage and other severe chronic diseases; (2) disturbances of consciousness, cognitive impairment, and language dysfunction; (3) a history of schizophrenia; (4) a history of drug abuse; (5) severe mental illness; (6) serious chronic illnesses that could lead to impaired consciousness; and (7) eye disease with low vision.

### 2.1. Data Analysis and Statistics

The qualitative variables were described by absolute and relative frequencies, and the chi-square test was used to analyse the association between the groups and the studied characteristics. The significance level adopted was 95%, and the statistical program used was the Data Analysis and Statistical Software for Professionals (Stata) version 11.0®.

### 2.2. Ethical Committee

The present study was submitted and approved by the Ethical and Research Committee of the ABC Medical School based on Resolution Number 466 of the Brazilian National Health Council (Reg. Number 779.435). All patients signed an informed consent and received a copy of the term.

## 3. Results

Based on the inclusion and exclusion criteria, 50 adult PWE and 50 patients with heart disease (PWH) were enrolled in the present study. Among these research participants, 42 were male and 58 were female. No significant differences in gender (*p* = 0.224), educational level (*p* = 0.267), and reported skin color (*p* = 0.019) were found. The average age for the PWE group was 41.8 (18–65) years and for the PWH group was 57.8 (40–65) years. There was a significant difference between the groups (*p* < 0.0001), with centile 42.5 years (95% CI 33.5–52.5) for PWE and centile 60 years (95% CI 57–63) for PWH. The onset of the disease was earlier in the PWE group, with a mean age of 11.5 years, compared with a mean of 50 years for the PWH group (*p* < 0.0001).

Twenty-two percent of PWH and 18% of PWE reported that they were employed (*p* = 0.617) while 8% of PWH and 58% of PWE were unemployed (*p* < 0.0001). A total of 32% of PWE reported being dismissed from work as a result of their epilepsy, while 6% of PWH affirmed that they were dismissed because of their heart disease (*p* = 0.001). For the PWE, 22 (44%) reported not being hired by an employer due to the disease, while only two such reports were found among the PWH (*p* < 0.0001). Eight (16%) PWE and 32 (64%) PWH were retired at the time of the survey (*p* < 0.001). Five (62.5%) of PWE and 21 (65.62%) of PWH were over 60 years old. The mean age of the participants at the time of retirement was 48 years for those with heart disease and 43 years for those with epilepsy, which was not significantly different. Both groups reported not disclosing the disease to their employer, (*p* = 0.161). In addition, 38% of the PWE and 9% of the PWH did not disclose their condition to their colleagues (*p* = 0.026). The PWE were more like to report that the reason for nondisclosure was that they felt ashamed, with 16% of PWE and 2% of PWH (*p* = 0.014). The presence of a depressive disorder requiring medication was reported for 20 PWE and one with heart disease (*p* = 0.004). The results are shown in Table 1.

## 4. Discussion

Few studies have compared the occupational issues between PWE and PWH. Both diseases are chronic conditions that require medical treatment and work restrictions, with the potential for permanent disability, depending on their clinical severity [17, 18].

Employability is not only important for subsistence, but also an instrument for increasing self-esteem and including the individual in social groups, thereby, increasing the likelihood of overcoming social barriers imposed by the disease [6, 19]. The analysed groups showed important discrepancies regarding the ages, and the participants in the study group were younger than the control group; these factors bias the results, mainly regarding employability. But even with these vies, it is possible to note the high rate of labour impediments among participants with epilepsy. In the present study, a high unemployment rate was observed in the PWE group, surpassing 50%, and was significantly higher than the PWH group. In Brazil, the minimum age for retirement is 55 years for women and 60 years for men. The average age of the participants at the time of retirement in both groups was below the minimum limit. These data suggest that both conditions are related to premature withdrawal from the workforce.

The difference between the groups in terms of age and the number of retirees also reflects on the unemployment situation. PWE showed higher unemployment rates than PWH. Two explanations are possible. The first is that having epilepsy, which carries a greater burden of stigma and prejudice, makes it difficult to enter the labour market. This explanation comes from the finding that more PWE responded that they were not employed or that they were dismissed from their jobs because of their disease, compared to patients with heart disease. The second explanation is that the higher rate of disability retirement granted by the social security institute to PWH occurs because it is a disease that is easier to prove in clinical terms. Many of the clinical manifestations of epilepsy are subjective, which makes it difficult to prove the disease in medical-expert evaluations. The literature shows that PWE have twice the rate of unemployment when compared to people without epilepsy [20]. Multiple other causes are associated with the greater tendency to be unemployed, including mental disorders [10, 21–24], low qualification, and lower educational level. In this study there were no differences between the groups regarding the educational level, but the participants' qualifications were not evaluated.

A study conducted in Brazil in 2002 evaluated the quality of life of 193 people aged 18 to 59 years who were diagnosed with epilepsy. The life area considered most compromised by the respondents was work related (31.29%), followed by leisure, physical health, and social relationships. Of the total analysed, 69.4% did not work, and 30% of these affirmed that their lack of work was due to epilepsy [25]. This finding is similar to that observed in the present study (58% unemployed).

PWE has a higher rate of depressive disorders when compared to those with depression being one of the most frequently reported comorbidities by PWE [26]. In our study, 20% of respondents with epilepsy reported using a pharmaceutical treatment to control depression. This finding was lower than the 11% to 60% reported by de Boer et al., 2008 [27]. The systematic review and meta-analysis showed that prevalence of active depression in PWE ranged from 13.2% to 36.5% [28].

The result of our study showed a low frequency of depression in group of heart failure, a result that is very different from the literature. Study evaluated depression in patients with heart failure and concluded that 21.5% met the criteria for active depression, varying from 19.3% for diagnoses by interviews and 33.6% by applying a questionnaire [29].

In 1967, “The Employable Epileptic” was published in Canada, in which it was argued that the major problems of employability of PWE were lack of knowledge about the nature of epilepsy and the effects of recent medical advances. After 35 years, the same arguments were repeated in a publication of the “Epilepsy Foundation of America,” reiterating that the main barrier in the occupation of the job market is fear [30]. This problem may reflect the higher rate of dissatisfaction of people with epilepsy and, consequently, higher rate of depression.

The PWE in our study stated that they hid the disease from employers and coworkers because they felt ashamed of the disease. The PWH also stated that they hid the disease from the employers, but not from their coworkers, and did not feel ashamed of the disease. These data reaffirm the thesis that epilepsy is still considered stigmatizing, as described in the literature [27, 31].

## 5. Conclusion

Compared to PWH, PWE tend to be unmarried, with a higher rate of unemployment, experience discrimination in seeking and in maintaining employment, and tend to feel ashamed of the disease at work and in their social life. The PWE put among their priorities the control of their seizures so that they can enter the labour market. However, it is not the seizures control failure that is the limiting factor. The simple declaration of being PWE works as a social barrier, often an insurmountable one. This study was conducted to evaluate the employability conditions of PWE compared to another chronic, often disabling, morbid condition, such as heart disease. The results showed that being a person with a chronic disease does not necessarily result in stigma and prejudice, but PWE specifically experience stigma and prejudice. The authors suggest that further studies should be performed with a larger sample of participants and with matching between the groups studied, especially in relation to age. The age discrepancy between groups was an important limiting factor of this study. However, despite the limited number of participants and the age difference between the groups, there is no impediment in stating that people with epilepsy show high rates of unemployment, depression, and stigma.

## Figures and Tables

**Table 1:** Distributions of the comparative data between groups.

	PWE	PWH	*p* value
Sex			
Male	18 (36%)	24 (48%)	0.224
Female	32 (64%)	26 (52%)	
Age	41,8 (18–65)	57,9 (40–65)	<0.0001
Skin color			
White	24 (48%)	37 (74%)	0.019^*∗*^
Black	3 (6%)	3 (6%)	
Brown	23 (46%)	10 (20%)	
Schooling			
Basic	4 (8%)	1 (2%)	0.267
Incomplete middle school	14 (28%)	16 (32%)	
Complete middle school	09 (18%)	7 (14%)	
Incomplete high school	1 (02%)	1 (02%)	
Complete high school	20 (40%)	17 (34%)	
Complete higher education	0	5 (10%)	
Incomplete higher education	2 (4%)	3 (6%)	
Employment status			
Employed	9 (18%)	11 (22%)	0.617
Retired	8 (16%)	32 (64%)	<0.0001^*∗*^
Sick leave	3 (6%)	0	0.079
Fired due to disease	16 (32%)	3 (6%)	0.001^*∗*^
Unemployed	29 (58%)	4 (8%)	<0.0001^*∗*^
Not hired due to illness	22 (44%)	2 (4%)	<0.0001
No disclosure of disease to employer	23 (46%)	30 (60%)	0.161
No disclosure of disease to colleagues	19 (38%)	9 (18%)	0.026^*∗*^
Feel ashamed to have the disease	8 (16%)	1 (2%)	0.014^*∗*^
Comorbidities	15 (30%)	16 (32%)	0.829
Depressive disorder	10 (20%)	1 (2%)	0.004^*∗*^

^*∗*^Pearson “Chi^2^” – *p* value < 0,05; PWE: patient with epilepsy; PWH: patient with heart disease.

## Abbreviations

AEM: Medication antiepileptic
PWE: People with epilepsy
PWH: Patients with heart disease.
